# Biodegradation of crude oil by individual bacterial strains and a mixed bacterial consortium

**DOI:** 10.1590/S1517-838246120131276

**Published:** 2015-06-01

**Authors:** Santina Santisi, Simone Cappello, Maurizio Catalfamo, Giuseppe Mancini, Mehdi Hassanshahian, Lucrezia Genovese, Laura Giuliano, Michail M. Yakimov

**Affiliations:** 1 Institute for Coastal Marine Environment, National Counsel of Research, Messina, Italy, Institute for Coastal Marine Environment, National Counsel of Research, Messina, Italy.; 2 Università degli Studi di Messina, School in Biology and Cellular Biotechnology, Faculty of Sciences, University of Messina, Messina, Italy, School in "Biology and Cellular Biotechnology", Faculty of Sciences, University of Messina, Messina, Italy.; 3 Università degli Studi di Catania, Department of Industrial Engineering, University of Catania, Catania, Italy, Department of Industrial Engineering, University of Catania, Catania, Italy.; 4 Shahid Bahonar University of Kerman, Department of Biology, Faculty of Sciences, Shahid Bahonar University of Kerman, Kerman, Iran, Department of Biology, Faculty of Sciences, Shahid Bahonar University of Kerman, Kerman, Iran.

**Keywords:** *Alcanivorax*, *Pseudomonas*, *Rhodococcus*, bioremediation, bioaugmentation

## Abstract

Three bacterial isolates identified as *Alcanivorax borkumensis* SK2, *Rhodococcus erythropolis* HS4 and *Pseudomonas stutzeri* SDM, based on 16S rRNA gene sequences, were isolated from crude oil enrichments of natural seawater. Single strains and four bacterial consortia designed by mixing the single bacterial cultures respectively in the following ratios: (*Alcanivorax: Pseudomonas*, 1:1), (*Alcanivorax*: *Rhodococcus,* 1:1), (*Pseudomonas*: *Rhodococcus*, 1:1), and (*Alcanivorax*: *Pseudomonas*: *Rhodococcus*, 1:1:1), were analyzed in order to evaluate their oil degrading capability. All experiments were carried out in microcosms systems containing seawater (with and without addition of inorganic nutrients) and crude oil (unique carbon source). Measures of total and live bacterial abundance, Card-FISH and quali-, quantitative analysis of hydrocarbons (GC-FID) were carried out in order to elucidate the co-operative action of mixed microbial populations in the process of biodegradation of crude oil. All data obtained confirmed the fundamental role of bacteria belonging to *Alcanivorax* genus in the degradation of linear hydrocarbons in oil polluted environments.

## Introduction

Petroleum hydrocarbons are the most widespread contaminants within the marine environment. Pollution by hydrocarbons in marine environments may be the consequence of various natural (natural seepages) and/or anthropogenic activities (discharge during tanks and/or ships transportation and/or pipeline failures) as well as the chronic pollution (ships, harbours, oil terminals, freshwater run-off, rivers and sewage systems).

The "fate" of petroleum in the sea water largely depends on mechanical (wave, wind), physical (temperature, UV) and chemical (pH, dissolved oxygen and nutrient concentration) factors which may differently influence its natural transformation (oil weathering) and bio-degradation (Nikolopoulou and Kalogeraki, 2010). At an early stage light fractions of oil are naturally removed; mostly by evaporation, thence by photo-oxidation and by geo-chemicals reactions. Heavy fractions are instead dispersed or dissolved and only a small portion may be removed by the process of biodegradation. Although chemical-physical phenomena play an important role in the process of oil detoxification, the ultimate and complete degradation is mainly accomplished by marine microflora, dominant bacteria in this role (Della Torre *et al.*, 2012).

As reported in different studies, a wide variety of marine bacteria are known to degrade petroleum hydrocarbons, and those, distributed over several (sub)phyla (α-,β-, and γ-Proteobacteria; Bacteroidetes/Chlorobi group) have been described so far (Rooling *et al.*, 2004; Cappello *et al.*, 2007).

In the natural environment, biodegradation of crude oil involves a succession of species within the consortia of the present microbes (Alkatib *et al.*, 2011). Indeed, since a single species can metabolize only a limited range of hydrocarbon substrates, a consortium of many different bacterial species, with broad enzymatic capacities, is usually involved in oil degradation (Rooling *et al.*, 2002). Although some bacteria, belonging to *Pseudomonas* (Das and Chandar, 2011) and *Rhodococcus* genera (Hassanshahian *et al.*, 2010 and 2012) have shown able to degrade hydrocarbons (Teramoto *et al.*, 2010), in marine environments the key micro-organisms in the bio-degradation process has been identified as bacteria related to *Alcanivorax* genus (Yakimov *et al.*, 2007; Cappello and Yakimov 2010).

On the above mentioned basis, bioremediation techniques have been developed and improved for cleaning up oil-polluted marine environments as an alternative to chemical and physical techniques (Alkatib *et al.*, 2011). Bioremediation can be described as the conversion of pollutants (hydrocarbons) by micro-organisms (bacteria) into energy, cell mass and biological waste products (Nikolopoulou and Kalogeraki, 2010). Nevertheless, the rates of uptake and mineralization of many organic compounds (hydrocarbons) by bacteria in polluted seawater is limited due to the poor availability of nitrogen and phosphorus (Yakimov *et al.*, 1998; Kasai *et al.*, 2002a, b; Cappello and Guglielmino, 2006; Cefalì *et al.*, 2002). For that reason, in the application of biostimulation techniques the growth of oil-degrading bacteria can be strongly enhanced by fertilization with inorganic nutrients (Nikolopoulou and Kalogeraki, 2010).

In order to elucidate the cooperative action of mixed microbial populations in the biodegradation of crude oil, we have built up artificial consortia made up of two/three bacteria. By using these consortia, we have been able to investigate the capability of efficient biodegradation of crude oil could be accomplished by the mixed populations. All experiments have been carried out into microcosms systems containing seawater (with and without inorganic nutrients); oil has been used as the only carbon source.

The knowledge of the efficiency and the activities of bacteria in oil-polluted sites may be helpful for the bioremediation of oil spills, since human action, by using specific microbial consortia, can be planned in order to clean up oil pollution (Denaro *et al.*, 2005).

## Material and Methods

### Bacterial strains

**Figure 1:**
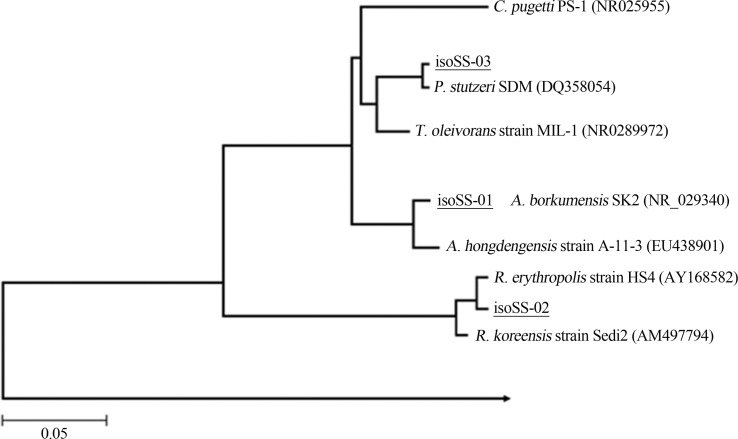
Phylogenetic tree based on 16S rRNA gene sequences for bacterial strains (isolates isoSS-01, -02 and -03) used in this study. Percentages of 100 bootstrap resampling that supported the branching orders in each analysis are shown above or near the relevant nodes. The tree was rooted and outgrouped (black arrow) by using the 16S rRNA sequences of *Methanococcus jannaschii* (M59126). Evolutionary distance is indicated by vertical lines; each scale bar length corresponds to 0.05 fixed point mutations per sequence position.

Three bacterial strains named isoSS-01, corresponding to *Alcanivorax borkumensis* strain SK2^T^ (Genbank accession number Y12579; =DSM 11573^T^; 99%), isoSS-02 (*Rhodococcus erythropolis* HS4*;* Genebank accession number AY168582; 99%) and isoSS-03 (*Pseudomonas stutzeri* SDM; Genebank accession number DQ358054; 98%) were used in all the experiments (Fig. 1). Strain isoSS-01 belong to a collection of hydrocarbon-degrading bacteria hold at IAMC-Messina, strains isoSS-2 and iso-SS03 were isolated from natural seawater from crude oil enrichments in previously research. All strains used in this study were isolated from natural seawater from crude oil enrichments.

### Analysis of 16S rRNA genes

Total DNA extraction of bacterial strains was performed by the MasterPure Complete DNA&RNA Purification Kit (Epicenter, Biotechnologies, Madison, WI) in accordance with manufacture's protocol. The 16S rDNA loci were amplified using 1 primer pair: the 27F (5′-AGAGTTTGATCCTGGCTCAG-3′, Lane, 1991) primer and the 1492R (5′ TACGGYTACCTTGTTACGACT-3′, Lane, 1991) universal primer. PCR (polymerase chain reaction) was carried out in 50 μL of reaction mixture containing 1× reaction buffer, 1× solution Q (both from QIAGEN), 1 μM of each primer, 200 μM dNTP (Gibco), 1 μL of template and 2.5 U of Qiagen *Taq* polymerase. The PCR reaction was carried out in Mastercycler Gradient (Eppendorf); the PCR conditions were as follows: 95 °C for 5 min (1 cycle); 94 °C for 1 min, 50 °C for 1 min and 72 °C for 2 min (35 cycles); with a final extension step at 72 °C for 10 min. PCR products were sequenced using Macrogen Service (Macrocen, Korea). The analysis of the sequences (1400 bp of average length) was performed as previously described by Yakimov *et al.* (2005). The sequences similarity of individual inserts was analysed by the FASTA program Nucleotide Database Query available through the EMBL-European Bioinformatics Institute. The phylogenetic affiliation of the sequenced clones, was performed as described by Yakimov *et al.* (2006).

### Growth conditions

Started cultures were prepared by inoculating one loop of microbial cells into 10 mL of ONR7a mineral medium based on the composition of seawater was used in this study (Dyksterhouse *et al.*, 1995). Nitrogen was provided in the form of NH_4_Cl, and was provided in the form of Na_2_HPO,. ONR7a contained (per liter of distilled or deionized water) 22.79 g of NaCl, 11.18 g of MgCl_2_*6H_2_O, 3.98 g of Na_2_SO_4_, 1.46 g of CaCl_2_, - 2H_2_O, 1.3 g of TAPSO {3-[N-tris(hydroxymethyl) methylamino]-2-hydroxypropanesulfonic acid}, 0.72 g of KCl, 0.27 g of NH_4_Cl, 89 mg of Na_2_HPO_4_ * 7H_2_O, 83 mg of NaBr, 31 mg of NaHCO3, 27 mg of H_3_BO_3_, 24 mg of SrCI*6H_2_O, 2.6 mg of NaF, and 2.0 mg of FeCl_2_*4H_2_0. To prevent precipitation of ONR7a during autoclaving, three separate solutions were prepared and then mixed together after autoclaving when the solutions had cooled to at least 50 °C; one solution contained NaCI, Na_2_SO_4_, KCl, NaBr, NaHCO_3_, H_2_BO_3_, NaF, NH_4_Cl, Na_2_HPO_4_, and TAPSO (pH adjusted to 7.6 with NaOH), the second solution contained MgCl_2_, CaCl_2_, and SrCI, (divalent cation salts), and the third solution contained FeCl_2_; 0.1% (w/v) sterile tetradecane (C_14_H_30_, Sigma-Aldrich, Milan, Italy) was used as only energy and carbon source. After growing in a rotary shaker (New Brunswick C24KC, Edison NJ, USA; 150 rpm) at 25 °C for two days, 500 μL of the seed culture broth were transferred into a 250 mL Erlenmeyer flask containing 100 mL of ONR7a medium supplemented with 1% (w/v) sterile tetradecane. The culture was incubated in a rotary shaker (New Brunswick C24KC, Edison NJ, USA; 150 xg) at 25 °C for 5 days.

### Consortia

**Figure 2:**
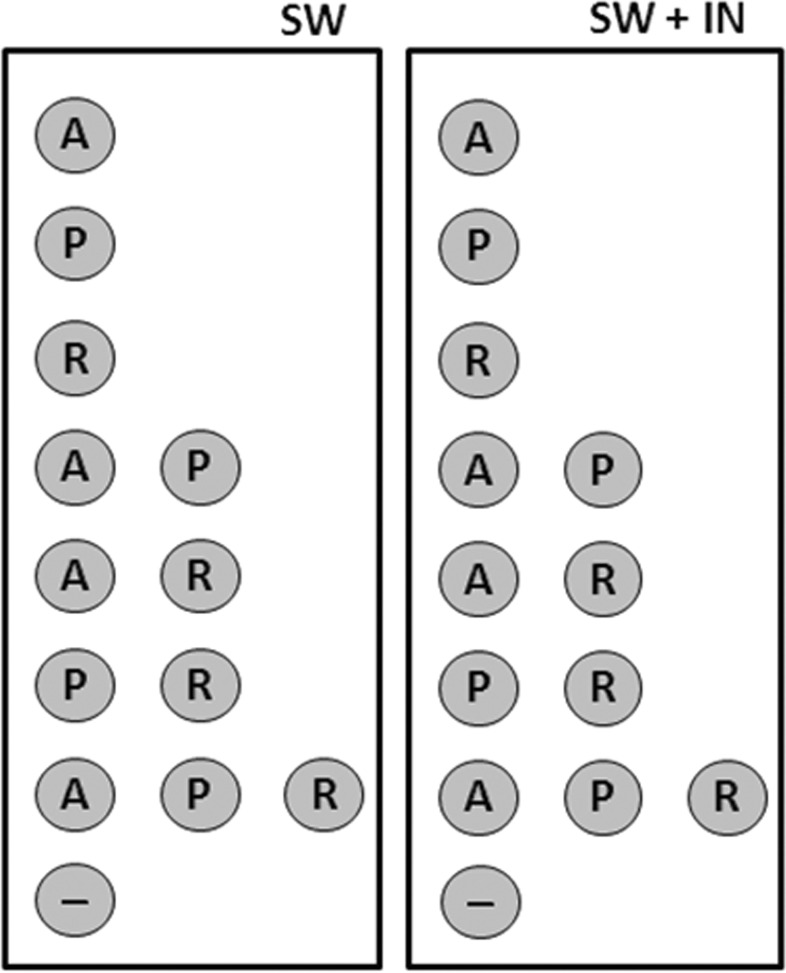
Schematic representation of microbial consortia and experimental microcosms carried out in this study. A, isolate isoSS-01, (A*. borkumensis* SK2); P, isolate iso-SS-03 (*Ps. stutzeri* SDM); R, isolate iso-SS-02 (*R. erythropolis* HS4); -, negative abiotic control.

At the beginning (T_0_) of the experiments selected microorganisms (isoSS-01, *A. borkumensis* SK2^T^; iso-SS-02, *R. erythropolis* HS4 and iso-SS-03 *Ps. stutzeri* SDM) were added at a final density of 10^5^ cell mL^−1^, in experimental microcosms. Schematic representation of microbial consortia used in this study is indicated below (Fig. 2).

### Experimental set-up of microcosms systems

The microcosms systems were performed in 250 mL sterilised Erlenmeyer flasks. Microcosms were incubated at 22 ± 1 °C for 15 days with shaking (100 *g*). All experiments were carried out in triplicate.

Two different series of experimentations were carried out. In the first experiment (identified as "SW") bacterial cultures were carried out in natural seawater sterilized by filtration through a 0.2-μm syringe filter (Sartorius); in the second experiment (identified "SW+IN") cultures were carried out in sterile natural seawater with addition of inorganic nutrients to reach higher concentrations than those obtained in natural water (final concentrations: KH_2_PO_4_0.077 g L^−1^, NH_4_Cl 0.2 g L^−1^and NaNO_3_0.1 g L^−1^). Microcosms untreated (no bacteria inoculation) were used in each experiment series as negative (abiotic) control. Crude oil was added in all experimentation.

At the beginning (T_0_) of the experiments, 1000 ppm of sterile crude oil (Arabian Light Crude Oil; ENI Technology S.p.A.) were added into SW and SW+IN microcosms. Crude oil was introduced, in microcosm systems, after physical weathering (100 × g, 25 °C for 48 h); crude oil was supplemented with 0.1% (v/v) of squalene (C_30_H_50_, Sigma-Aldrich, Milan) as internal spike for measure of bio-degradation rate.

### Sampling strategy and parameters assayed

At the beginning (T_0_) and at the end (T_15_) of the experimental period, sub-samples of each bacterial cultures were taken aseptically. Measures of direct bacterial count (DAPI), microbial viability (Live/Dead staining) and microbial activity (Card-FISH) were carried out. Measure of oil degradation was carried out as well. All experiments were carried out twice and all parameters detected were measured three times.

### Total bacterial abundance (DAPI count)

After a short-time (30′) ultrasonic treatment (*Ultrasonic Bath Branson 1200*, Branson, USA), the total bacterial cell counts were performed by DAPI (4′,6-diamidino-2- phenylindole 2HCl, Sigma-Aldrich S.r.L., Milan, Italy) staining on samples fixed by formaldehyde (2% final concentration), according to Porter and Feig (1980) and Cappello *et al.*, 2012. Slides were examined by epifluorescence by using Axioplan 2 Imaging (Zeiss; Carl Zeiss Inc., Thornwood, N.Y.) microscope. Results were expressed as number of cells mL^−1^.

### Determination of living and dead bacteria

Living and dead bacteria (L/D) were enumerated after staining with the Live/Dead (BacLight bacterial Viability Kit (Invitrogen Corp; Molecular Probes, Inc Eugene, OR, USA). The above mentioned method allowed discrimination, within the total bacterial community, of the living cells, labelled by SYTO 9 and green-fluorescing, from the dead ones, labelled by propidium iodide and red-fluorescing (Zampino *et al.*, 2004). Cell counts, performed by an Axioplan epifluorescence microscope (Zeiss; Carl Zeiss Inc., Thornwood, N.Y., USA) equipped with a 100 W Hg lamp using fluorescein (BP 450–490; FT 510; LP 520) and rhodamine (BP 546/12; FT 580; LP 590) filter sets (for live and dead cells, respectively). Data obtained were reported as the mean value of (living and dead) cells mL^−1^.

### Card-FISH

Card-FISH analysis was carried out according to protocol developed by Pernthaler *et al.* (2002). Aliquot of 1 mL of bacterial culture was filtered on 0.22 μm polycarbonate membranes (diameter 25 mm) by using a vacuum filtration device (Millipore, Milan, Italy). Filters for Card-FISH counts were embedded in low-gelling point (0.1% agarose, Sigma-Aldrich, Milan), dried at 37 °C for 20 min, and dehydrated with 95% ethanol. The bacteria on the polycarbonate membrane were then permeabilized by lysozyme (solution (EDTA 0.05 M; 1 M Tris-HCl, pH 8.0; MilliQ water and 10 mg mL^−1^ lysozyme) for 60 min at 37 °C and in some cases a treatment with achromopeptidase (60 U, 0.01 M NaCl, 0.01 M Tris-HCl [pH 8.0]) was performed. Filters were incubated at 37 °C for 30 min and hybridized with oligonucleotide probes modified at the 5′ end with horseradish peroxidase (HRP). Probes used in this work are listed in the Table 1.

**Table 1:** Oligonucleotide probes used in Card-FISH for this study.

Probe	Sequence (5′ to 3′) of probe	Specificity	Source
NON-Eub338	ACA TCC TAC GGG AGG C	Negative Control	(Wallner *et al*., 1993)
Eub338	GCT GCC TCC CGT AGG AGT	Domain Bacteria	(Amann *et al*., 1990)
Alk	CG ACG CGA GCT CAT CCA TCA	Alcanivorax genus	(Karner and Fuhrman, 1997)

After the hybridization and amplification steps, slides were examined by an Axioplan epifluorescence microscope (Zeiss; Carl Zeiss Inc., Thornwood, N.Y., USA) equipped with an appropriate filter sets for Card-FISH. Before counting, the slides were stored at −20 °C for several days without any loss of fluorescence intensity. Cell counts were reported as the mean value of cells mL^−1^.

### Hydrocarbon analysis

The composition of the Total Extracted and Resolved Hydrocarbons and their derivates (TERHCs) were analysed by high-resolution GC-FID (*DANI Master GC* Fast Gas Chromatograph System, DANI Instruments Sp.A., Milan). After acidification, TERHCs from samples were extracted at room temperature on a shaking table by using dichloromethane (CH_2_Cl_2_, Sigma-Aldrich, Milan; 10% v/v). This procedure was repeated three times, and the CH_2_Cl_2_ phase was combined and treated with sodium sulfate anhydrous (Na_2_SO_4,_ Sigma-Aldrich, Milan) in order to remove any residual water (Ehrhardt *et al.*, 1991; Wang *et al.*, 1998; Dutta and Harayama 2001; Denaro *et al.*, 2005). Extracts were concentrated by rotary evaporation (Rotavapor model R110; Büchi Labortechnik AG, Switzerland) at room temperature (< 30 °C), followed by evaporation under a stream of nitrogen and taken up into a solution containing heptamethyl-nonane as an internal standard (79 μg mL^−1^). Indices selected for this study were: *n*-C17/Pristane (*n*C17/Pr), *n*-C18/Phytane (*n*C18/Ph) in order to evaluate the relative biodegradation of *n*-alkanes.

### Biodegradation efficiency (BE) of TERCHs

The degradation of TERCHs was expressed as the percentage of TERCHs degraded in relation to the amount of the remaining fractions in the appropriate abiotic control samples. The biodegradation efficiency (BE), based on the decrease in the total concentration of hydrocarbons, was calculated by using the expression described by Michaud *et al.*, 2004:

100-(As*100/Aac)

where As = total area of peaks in each sample, Aac = total area of peaks in the appropriate abiotic control, BE (%) = Biodegradation efficiency.

### Statistical analysis and nMDS

The experimental data are presented in terms of arithmetic averages of at least three replicates and the standard deviations are indicated by error bars. The non-metric multi-dimensional (nMDS) scaling plot were done using PAST (PAlaeontological STatistics Software ver. 1.88; Hammer *et al.*, 2001).

## Results

### Total bacterial abundance (DAPI count)

**Figure 3:**
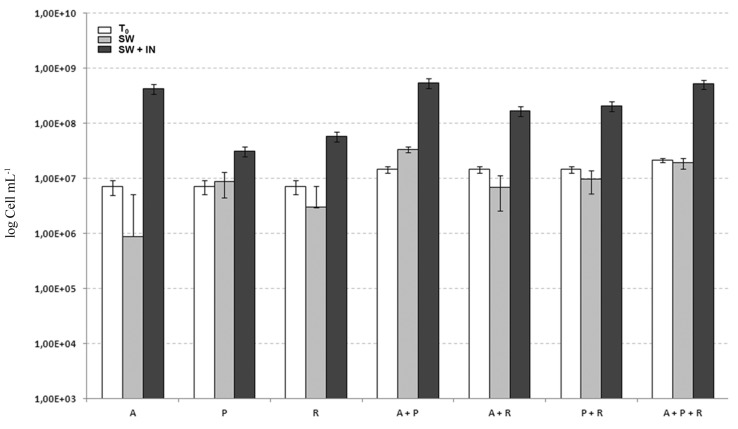
Measure of bacterial abundance by direct DAPI count in cultures obtained in seawater without addition of inorganic nutrients (grey bars) and with addition of inorganic nutrients (dark grey bars). A, iso-SS-01 (*Alcanivorax borkumensis* SK2^T^); P, iso-SS-02 (*Pseudomonas stuzteri* SMD) and R, iso-SS-03 (*Rhodococcus erythropolis* HS4).

After 15 days of cultivation, the bacterial abundance was measured by direct DAPI count; and data obtained were compared with the quantity of cells present at the beginning of the experimental period (T_0_). The data obtained showed, how in seawater added with inorganic nutrients it was possible to observe a general increase of microbial abundance (systems "A", "A + P", "A + R," "P + R", and "A + P + R ") with mean values of 10^8^ cell mL^−1^. In cultures performed using seawater (without inorganic nutrients), bacterial abundance present, at the end of experimental period, mean values of 10^6^ cell mL^−1^ (systems "P", "R", "A + P" and "A + P + R"); in microcosms indicated as "A", "A + R", and "P + R") values of ~10^5^ cell mL^−1^ were observed (Fig. 3).

### Determination of living and dead bacteria

**Figure 4:**
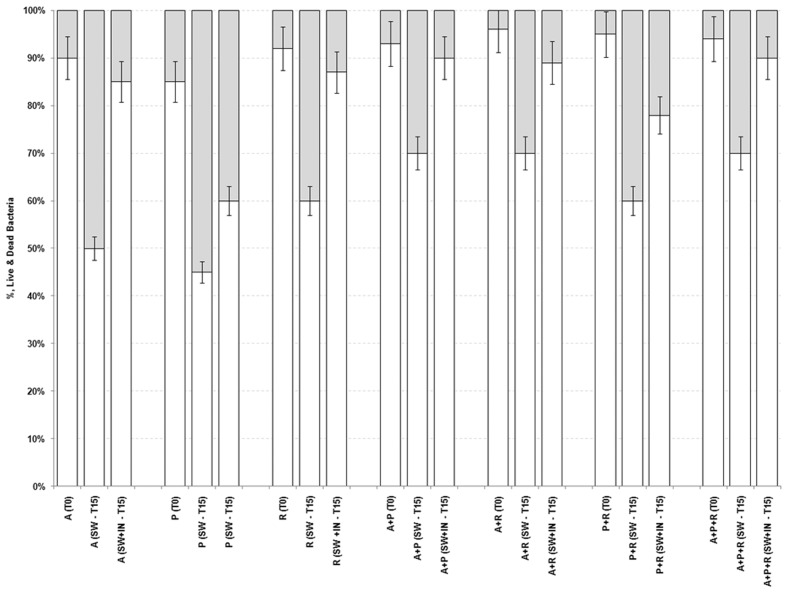
Results of living and dead bacteria (L/D) in cultures obtained in seawater with and without addition of inorganic nutrients. A, iso-SS-01 (*Alcanivorax borkumensis* SK2^T^); P, iso-SS-02 (*Pseudomonas stuzteri* SMD); R, iso-SS-03 (*Rhodococcus erythropolis* HS4); withe bars, live cells; grey bars, dead cells.

Data of living and dead bacteria (L/D) enumerated using the Live/Dead staining are showed in Figure 4.

The results obtained after 15 days of cultivation shown as the vital bacterial fraction, present in microcosm performed in seawater with addition of inorganic nutrients, was greater than that observed in the microcosms performed in sea water. In particular in microcosms indicated as SW the percentage of dead cells was about four or six times greater than the initial time.

### Card-FISH

The qualitative measure of microbial abundance, into the experimental systems named "A+P" and "A+R", was carried out by using the card-FISH method. Values of abundance of cells hybridized using probes for Eubacteria (EUB338) resulted to be similar to the values obtained from the measure of total bacterial abundance (DAPI count) in the same conditions.

**Figure 5:**
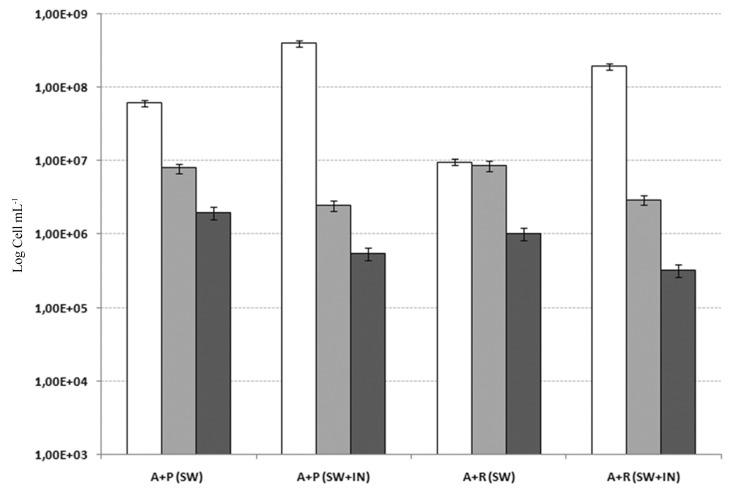
Bacterial abundance detected by card-FISH in bacterial consortia ("A+ P" e "A + R") during growth in natural seawater in absence (SW) and presence (SW + IN) of inorganic nutrients. Results obtained with hybridization with probe for Eubacteria and *Alcanivorax* sp. are indicated, respectively, with grey and dark grey bars. Bacterial total count (DAPI) was indicated in white bars.

Data obtained put in evidence as almost total cells of experimentations carried out with seawater without inorganic nutrients were hybridized by probes for Eubacteria. The same result was not obtained during experimentations carried out with seawater added with inorganic nutrients (in such a case a number of cells of a lower logarithmic order has been obtained). The data obtained showed as the quantity of cells of *Alcanivorax borkumensis* (in "A + P", SW + IN; "A + R", SW and "A+ R", SW + IN systems) present values lower (of a logarithmic order) those obtained in total cells (Fig. 5).

### Rate of degradation of n-alkanes

**Figure 6:**
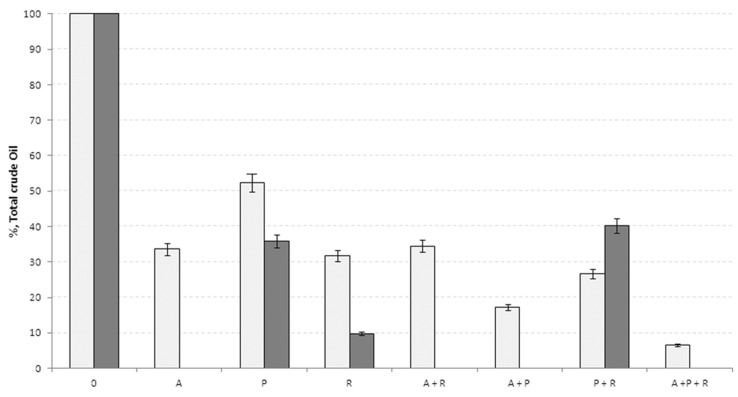
Relative values of major TERHC fractions of Arabian Light Crude Oil detected in SW and SW+IN cultures after 15 days of incubation; data expressed as the percentages compared to negative abiotic control (0). A, *Alcanivorax borkumensis* SK2; P, *Pseudomonas stuzteri* SMD; R, *Rhodococcus erythropolis* HS4. Experimentations carried out in natural seawater in absence (SW) and presence (SW + IN) of inorganic nutrients were indicated, respectively, with grey and dark grey bars.

The percentage degradation of n-alkanes (C_12_–C_30_) present in the crude oil was calculated by comparison of the gas chromatograms of the non degraded (abiotic) control and the degraded sample for each experimental conditions (Table 2 and Fig. 6).

**Table 2:** Percentage of *n*-alkane degradation in crude oil by strains in this study after 15 days of incubation at 22 ± 1 °C with shaking (100 *g*). Table Top, bacterial culture s in natural sea water; Table Lower, Bacterial cultures performed in natural seawater with inorganic nutrients. A, *Alcanivorax borkumensis* SK2; P, *Pseudomonas stuzteri* SMD; R, *Rhodococcus erythropolis* HS4.

n-alkanes	Natural sea water						
	
	A	P	R	A+P	A+R	P+R	A+P+R
C_12_	95	80	82	86	100	90	99
C_13_	69	52	57	68	94	92	96
C_14_	56	45	48	52	74	94	77
C_15_	42	42	49	51	82	94	92
C_16_	46	46	52	57	81	93	92
C_17_	51	48	53	57	79	91	92
C_18_	52	49	56	57	81	89	93
C_19_	63	63	68	65	82	88	94
C_20_	60	59	64	62	78	86	93
C_21_	66	67	70	68	79	82	93
C_22_	68	69	72	67	80	79	94
C_23_	77	77	80	74	84	79	95
C_24_	66	68	71	62	78	69	94
C_25_	74	75	78	70	82	66	95
C_26_	75	77	79	71	84	66	95
C_27_	75	76	78	70	83	53	95
C_28_	75	76	79	70	84	47	95
C_29_	73	73	77	67	83	37	95
C_30_	76	77	81	72	87	31	96

n-alkanes	Natural sea water + inorganic nutrient						
	
	A	P	R	A+P	A+R	P+R	A+P+R

C_12_	100	87	100	100	100	100	100
C_13_	100	88	97	100	100	100	100
C_14_	100	89	95	100	100	100	100
C_15_	100	82	91	100	100	100	100
C_16_	100	84	95	100	100	90	100
C_17_	100	85	95	100	100	81	100
C_18_	100	83	94	100	100	94	100
C_19_	100	83	90	100	100	80	100
C_20_	100	82	92	100	100	80	100
C_21_	100	81	90	100	100	77	100
C_22_	100	81	92	100	100	72	100
C_23_	100	83	90	100	100	71	100
C_24_	100	76	87	100	100	63	100
C_25_	100	77	90	100	100	68	100
C_26_	100	77	86	100	100	70	100
C_27_	100	75	87	100	100	71	100
C_28_	100	75	86	100	100	69	100
C_29_	100	75	81	100	100	71	100
C_30_	100	77	87	100	100	62	100

During experimentations performed with natural seawater the condition identified as "A+P+R" showed a better rate degradation (~ 90%); also in system "A+R" in other conditions is possible to observe a degradation of almost all n-alkanes (rate of degradation > of 60%).

The data obtained show that, during growth in natural seawater added with inorganic nutrients, conditions "A", "R", "A+P", "A+ R" and "A+P+R" n-alkanes present in the crude oil were totally degraded; in contrast, conditions "P" and "P+R" present a low rate of degradation of n-alkanes.

**Figure 7:**
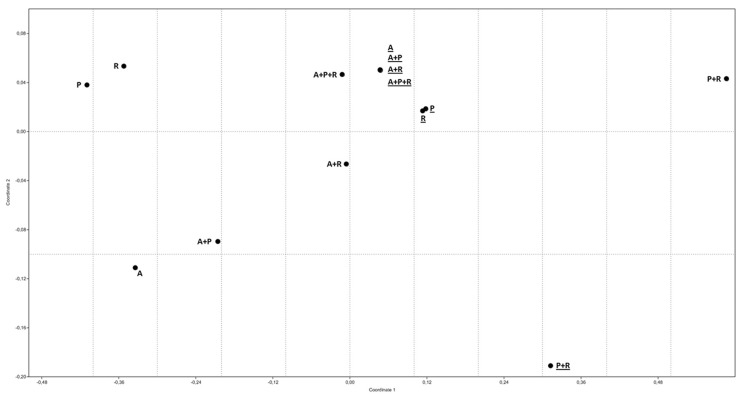
The non-metric multi-dimensional (nMDS) scaling plot related to the capability biodegradation of n-alkanes of different bacteria and consortia in study. A, *Alcanivorax borkumensis* SK2; P, *Pseudomonas stuzteri* SMD; R, *Rhodococcus erythropolis* HS4. Normal letter indicate the Natural Sea Water experimentation (SW), underlined letters indicate the Natural Sea Water + Inorganic Nutrients (SW + IN) experimentation.

**Figure 8:**
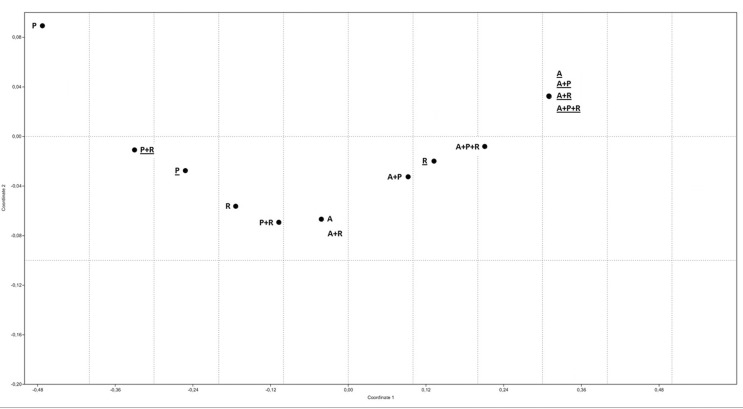
The non-metric multi-dimensional (nMDS) scaling plot related to the biodegradation efficiency (BE) of TERCHs of different bacteria and consortia in study. A, *Alcanivorax borkumensis* SK2; P, *Pseudomonas stuzteri* SMD; R, *Rhodococcus erythropolis* HS4. Normal letter indicate the Natural Sea Water experimentation (SW), underlined letters indicate the Natural Sea Water + Inorganic Nutrients (SW + IN) experimentation.

For all strains, n-alkanes with a medium length (C_12_–C_18_) were degraded to a greater extent (rate of degradation > of ~ 70%) than and long chains (C_19_–C_30_) because long-chain n-alkanes are solid and their low solubility inhibits degradation by bacteria (Figs. 7 and 8).

### Biodegradation efficiency (BE) of TERCHs

After 15 days of experimentation, measure of degradation of the TERHCs revealed as major rates of oil degradation are, in general, observed in systems carried out in natural seawater with inorganic nutrients (Table 3). In SW experiment the maximum rate of total oil degradation is observed in "A+P+R" (~ 97%) and "A+R" system (~ 83%). Other conditions present similar values. In system SW+IN the experimentations identified "A", "A+P", "A+R" and "A+P+R" the degradation of oil is total; values of ~ 90%, ~ 64% and ~ 30% of total oil degradation were observed for "P", "R" and "P+R" experiments (Fig. 6).

**Table 3:** Biodegradation efficiency (BE) of TERCHs. The experimental data are presented in terms of arithmetic averages.

Code	Natural sea water	Natural sea water+IN
A	64	100
P	48	64
R	64	90
A+R	66	100
A+P	83	100
P+R	73	60
A+P+R	93	100

## Discussion

The recovery of petroleum contaminated sites could be achieved by either physicochemical or biological methods. Due to negative consequences of the physicochemical approach, more attention is now given to the exploitation of biological alternatives (Okoh, 2006).

Biological treatments are having more importance, mainly because of the low environmental impact, the costs (in general cheaper than other cleanup technologies), the capability to destroy organic contaminants, and the possibility of beneficial use of treated sediments (Rulkens and Bruning, 2005). Different studies have shown better results using bioremediation strategies (Beolchini *et al.*, 2010; Rocchetti *et al.*, 2011, 2012).

In general, bioremediation is often based on *in-situ* stimulation of the microbial community (*biostimulation*) or amending the microbial community with an inoculum of hydrocarbon-degrading bacteria (*bioaugmentation*). In both cases, the successful result of bioremediation depends on appropriate hydrocarbon-degrading consortia and environmental conditions*.*

In this study we have analyzed the cooperative action of mixed microbial populations in the biodegradation of crude oil during different culture conditions. All data obtained confirmed the fundamental role of bacteria belonging to *Alcanivorax* genus in degradation of linear hydrocarbons in oil polluted environments. Indeed, all experimentations carried out in seawater (with or without inorganic nutrients) whit presence of *Alcanivorax* showed maximum rates of oil degradation.

Capability of *Alcanivorax* genus to use hydrocarbons as the only sources of energy and organic carbon was widely (Yakimov *et al.*, 1998; Scheiner *et al.*, 2006). Kasai (2002) and Cappello (2012) explain these characteristics in ability of this strain to produce a lipidic bio-surfactant that increases the bioaviable of contaminant and the ability to use this (Yakimov *et al.*, 1998; Scheiner *et al.*, 2006). *Alcanivorax borkumensis* SK2 surfactant propose as one of the most efficient of bacterial surfactants; the possible presence of this surfactant can justify an increase in the rates of degradation by both the bacteria that possible microbial consortia. This defines an increment of rates of degradation by both the bacteria and possible microbial consortia (Yakimov *et al.*, 1998; Scheiner *et al.*, 2006).

The presence of *Alcanivorax* in natural environment or enrichment by laboratory is generally combined with the presence of other bacterial strains, such as *Pseudomonas sp.* and *Rhodococcus sp.*, that participating in biodegradation phenomena. However, *Pseudomonas sp.* and *Rhodococcus sp.*, can not be classified such as hydrocarbonoclastic bacteria (Marine Obligate Hydrocarbonoclastic Bacteria, OMHCB; Yakimov *et al.*, 2007), but these are heterotrophic bacteria that participate in the biodegradation processes via "syntrophy metabolic"" in which the degradation of pollutant compounds takes place via a metabolic chain, in which the product of the catabolism of a bacterial species is identified as a source of carbon for metabolic another.

Analysis of microbial abundance in cultures in study showed, however, a divergence of the correlation between microbiological data and those of biodegradation. In experimentation carried out with *Alcanivorax* and *Pseudomonas* (system "A", "P" and "A+P") was possible to observe after 15 days to incubation in seawater with and without inorganic nutrients an increase of microbial biomass.

Data obtained during cultivation of *Rhodococcus erythropolis* (as single strain and/or as consortium) did not show, apparently, increment of microbial abundance. This condition may be due to an underestimation of the direct count (DAPI count) in cultures as result from an inefficiency of methodology used by us for the separation of microbial cells from oil remain (dislodging).

*Rhodococcus erythropolis* HS4, in presence of linear hydrocarbons, is usually produced trehalose lipids (Rapp, 1979); these molecules are composed of a disaccharide in combination with a long chain of fatty acids. The presence of these molecules define a general reduction of superficial (surface) tension such as increase of cellular hydrophobicity and consequently increase of bacterial tackiness.

Another important aspect was obtained to qualitative measures of microbial abundance. Card-FISH analysis carried out to estimate quantitative abundance of bacteria belonging to *Alcanivorax* genus in microbial consortia tested in this study. Therefore Card-FISH measures were realized for identified consortia A+P and A+R realized in seawater with and without inorganic nutrients. Hybridization with EUB-338 probe showed values similar to these obtained by direct DAPI count; for against assays carried out with ALK probe (specific probe to *Alcanivorax* genus) evidence as only the 15% of total cells were hybridized. This result can seem discordant with biodegradation results. However, it is important remember that the sample to Card-FISH was collected after 15 days of incubation, therefore is possible that the cells were collected in advance stationary phase and/or not more active. Supposing that the oil degradation process began early of the end of experiment, *Alcanivorax sp.*, dominant at the first experimental phase, tended to disappear or decrease once hydrocarbons have been degraded, while *Pseudomonas sp.* and *Rhodococcus sp.* cells could become dominant using metabolic compounds or cellular lysates like nutritional source.
